# Adolescent behavioural intentions: Secondary outcomes from a cluster randomized controlled trial of the Health4Life school-based lifestyle modification intervention

**DOI:** 10.17269/s41997-024-00955-w

**Published:** 2024-11-19

**Authors:** Siobhan O’Dean, Scarlett Smout, Matthew Sunderland, Tim Slade, Lauren A. Gardner, Cath Chapman, Louise Thornton, Bridie Osman, Emily Hunter, Lyra Egan, Maree Teesson, Nicola C. Newton, Katrina E. Champion

**Affiliations:** 1 The Matilda Centre for Research in Mental Health and Substance Use, The University of Sydney, Sydney, NSW Australia; 2 School of Medicine and Public Health, The University of Newcastle, Newcastle, NSW Australia; 3 Faculty of Medicine and Health, University of New South Wales, Kensington, Australia

**Keywords:** Adolescent, Health behaviour, Lifestyle, Intention, Schools, Adolescent, Comportements de santé, Mode de vie, Intention, Établissements scolaires

## Abstract

**Intervention:**

Health4Life: a school-based eHealth intervention targeting multiple health behaviour change (MHBC).

**Research question:**

Does Health4Life impact secondary outcomes of self-reported intentions regarding six lifestyle behaviours in adolescents (alcohol use, tobacco smoking, screentime, physical activity, discretionary beverage consumption, and sleep)?

**Methods:**

We implemented a cluster randomized controlled trial within secondary schools across three Australian states. Schools were randomly assigned (1:1) to receive either the Health4Life intervention, which consisted of a six-module, web-based program and accompanying smartphone app, or an active control (standard health education). Randomization was stratified by site and school gender composition (using Blockrand in R). All students aged 11–13 years who attended the participating schools and were fluent in English were eligible. Students completed self-report questionnaires at baseline, post-intervention, 12 months, and 24 months. Outcomes were intentions to try alcohol, try tobacco, reduce screentime, engage in physical activity on most or all days, swap discretionary beverages for water, and meet sleep guidelines. Mixed effects models estimated between-group differences in the outcomes over 24 months.

**Results:**

Immediately post-intervention, Health4Life significantly reduced intentions to try alcohol and tobacco and increased intentions for longer sleep and reduced screentime compared to control. Intervention effects on screentime intentions persisted at 12 months but not at 24 months. No lasting effects were observed on intentions for physical activity or discretionary beverage consumption.

**Conclusion:**

Health4Life shows promise in influencing adolescent intentions across various MHBC areas, especially immediately after intervention. However, further investigation is needed to sustain these intention changes beyond short term to facilitate behaviour change.

**Supplementary Information:**

The online version contains supplementary material available at 10.17269/s41997-024-00955-w.

## Résumé

**Intervention:**

Health4Life, une intervention de télésanté en milieu scolaire qui cible de multiples changements des comportements de santé.

**Question de recherche:**

Health4Life influence-t-elle les résultats secondaires des intentions autodéclarées d’améliorer 6 comportements liés au mode de vie des adolescents (consommation d’alcool, tabagisme, temps d’écran, activité physique, consommation de boissons discrétionnaires, sommeil)?

**Méthode:**

Nous avons appliqué un essai comparatif randomisé en grappes dans des écoles secondaires de trois États australiens. Les écoles ont reçu de façon aléatoire (1:1) soit l’intervention Health4Life, un programme en ligne en six modules accompagné d’une application pour téléphone intelligent, soit une intervention témoin active (le programme d’éducation sanitaire standard). La sélection aléatoire a été stratifiée (à l’aide de Blockrand in R) selon le lieu des écoles et leur équilibre entre garçons et filles. Étaient admissibles les élèves de 11 à 13 ans qui fréquentaient les écoles participantes et qui maîtrisaient l’anglais. Ces élèves ont rempli un questionnaire d’auto-évaluation au départ, après l’intervention, après 12 mois et après 24 mois. Les résultats étaient les intentions d’essayer l’alcool, d’essayer le tabac, de réduire le temps d’écran, de faire de l’activité physique tous les jours ou presque, de boire de l’eau au lieu de boissons discrétionnaires et de respecter les lignes directrices en matière de sommeil. Des modèles à effets mixtes ont permis d’estimer les écarts dans les résultats entre chaque groupe sur une période de 24 mois.

**Résultats:**

Tout de suite après l’intervention, Health4Life a significativement réduit les intentions d’essayer l’alcool et le tabac et accru les intentions de dormir plus longtemps et de réduire le temps d’écran comparativement à l’intervention témoin. Les effets de Health4Life sur l’intention de réduire le temps d’écran subsistaient à 12 mois, mais pas à 24 mois. Aucun effet durable n’a été observé sur les intentions liées à l’activité physique et à la consommation de boissons discrétionnaires.

**Conclusion:**

Health4Life est une intervention prometteuse pour ce qui est d’influencer les intentions des adolescents à l’égard de multiples changements des comportements de santé, surtout immédiatement après l’intervention. Une étude plus poussée est cependant nécessaire pour soutenir ces intentions par la suite afin de faciliter les changements de comportements.

## Introduction

Non-communicable diseases—including cardiovascular diseases, mental disorders, and cancer—are the leading causes of death and disability worldwide (GBD 2019 Diseases and Injuries Collaborators., 2019). In 2019, non-communicable diseases accounted for 63.8% of disability-adjusted life years globally (1.62 billion DALYs) (GBD 2019 Diseases and Injuries Collaborators, 2019). Lifestyle risk factors including smoking, substance use, physical inactivity, and poor-quality diets are leading risk factors for non-communicable diseases (Murray et al., 2020). These lifestyle risk factors typically emerge in adolescence where they can then become entrenched into adulthood (Due et al., 2011). A recent review estimated that investing in prevention of lifestyle risk factors in adolescence would generate a US$5 increase in human capital for every $1 spent (Watkins et al., 2019). Prevention of lifestyle risk factors not only reduces long-term risks of non-communicable disease, it is also associated with improved quality of life in the immediate term (Wu et al., 2017, 2019). Beyond adolescents’ immediate- and long-term health, improving adolescent health creates the healthiest possible start to life for their own children, an effect known as the “triple dividend” (Patton et al., 2016). As such, there is a strong economic and social imperative to invest in prevention in the critical window of adolescence.

The “Big 6” lifestyle risk factors—physical inactivity, poor diet, poor sleep, sedentary recreational screentime, alcohol use, and tobacco smoking—are highly prevalent and commonly co-occur among adolescents (Uddin et al., 2020). Around the world, the majority of adolescents do not meet recommended levels of physical activity and exceed recommended sedentary recreational screentime (van Sluijs et al., 2021). Insufficient sleep is common (Gariepy et al., 2020), and many consume processed, nutritionally poor diets with high added sugar, particularly through discretionary drinks including soft drinks, cordials, energy drinks, and sports drinks (Keller & Bucher Della Torre, 2015). Adolescent alcohol and tobacco smoking have decreased in most high-income countries between 1999 and 2014; however, since 2015, alcohol use has plateaued and even increased in some high-income countries (Ball et al., 2023). Adolescent tobacco smoking remains relatively low, with prevalence around 2–5% in Australia, New Zealand, England, and the United States (Ball et al., 2023). However, the long-term consequences of smoking are so impactful that even this low prevalence is likely to contribute substantially to the overall burden of disease (Murray et al., 2020). Evidence shows that these behaviours tend to cluster together, with more than 30% of adolescents worldwide engaging in three or more of these lifestyle risk behaviours, compounding their risk (Uddin et al., 2020). Thankfully, this clustering can also be positive, with evidence that improvements in one behaviour are associated with improvements in others (Prochaska et al., 2008).

There are several prevailing theoretical frameworks that outline pathways to behaviour change: the Theory of Reasoned Action (TRA), the Theory of Planned Behaviour (TPB), the Trans-Theoretical Model (TTM, also referred to as the Stages of Change Model), and the Self-Determination Theory (SDT) (Ryan & Deci, 2000; Taylor et al., 2006). While these frameworks differ in many ways, all argue that an individual must have an intention to change their behaviour for behaviour change to occur. Intentions themselves are mental plans to act in a certain way to achieve a desired outcome (e.g. “I will exercise 6 days a week to increase my physical fitness”). As such, intentions capture the motivational factors that influence behaviour, and moderate changes in intentions are associated with small to moderate changes in behaviour (Webb & Sheeran, 2006). Several studies have found that the constructs of behavioural intention are applicable to lifestyle behaviour changes, including alcohol use (Cooke et al., 2016), tobacco smoking (Webb & Sheeran, 2006), physical activity (Plotnikoff et al., 2013), and diet (Riebl et al., 2015).

Multiple health behaviour change (MHBC) interventions offer an efficient and cost-effective way to target several lifestyle risk factors simultaneously, capitalizing on evidence around behavioural clustering (Prochaska et al., 2008). Some studies have examined intervention effects on intentions to change a single or a subset of the Big 6 behaviours (Webb & Sheeran, 2006). Indeed, a recent umbrella review and meta-meta-analysis found that eHealth interventions are broadly effective at improving health behaviours (Singh et al., 2024). However, to date, no studies have examined whether behaviour MHBC interventions can impact the strength of intention to change behaviour across the Big 6 domains.

Health4Life is a school-based intervention targeting the Big 6 through both universal and targeted prevention components (Champion et al., 2021; Teesson et al., 2020). Health4Life was evaluated in a cluster randomized controlled trial (cRCT) with 71 schools across three Australian States (New South Wales [NSW]; Queensland [QLD]; Western Australia [WA]) from 2019 to 2022 (Champion et al., 2021; Teesson et al., 2020). The Health4Life intervention was informed by multiple behavioural theories, including SDT, which proposes a behaviour change pathway from “amotivation”—lacking an intention to change—to “motivation”—a sense of personal causation and increased intentionality around behaviours (Ryan & Deci, 2000; Teesson et al., 2020). SDT argues that the perceived value of a behaviour must increase to influence behavioural intentions (Ryan & Deci, 2000; Teesson et al., 2020). Health4Life leveraged social influence theory to target perceived value, using co-designed cartoon story-based lessons with characters the same age as students providing education around the benefits and harms associated with the Big 6 while concurrently targeting normative perceptions (Champion et al., 2020).

As the Health4Life trial tested a MHBC intervention spanning six lifestyle behaviours and mental health, it has numerous pre-registered primary and secondary outcomes (ANZCTR trial registration: ACTRN12619000431123). As such, the results of the Health4Life intervention against the pre-registered primary outcomes (six primary behaviour change outcomes for the Big 6 behaviours) and a number of secondary outcomes have been published elsewhere (Champion et al., 2023; O’Dean et al., 2023; Smout et al., 2024). In summary, the intervention increased knowledge about the Big 6, with effects persisting for up to 2 years, and showed short-term benefits for depression and psychological distress symptoms, but there were no significant between-group differences in the primary or secondary behaviour change outcomes (Champion et al., 2023; O’Dean et al., 2023; Smout et al., 2024). Even in the absence of immediate changes in behaviour, positive changes in behavioural intentions would suggest that the intervention lays a foundation for behaviour change. Moreover, examining the effects of Health4Life on intentions can give insight into areas that require further refinement or modifications to enhance the intervention’s efficacy in promoting behaviour change.

The present study aims to evaluate the efficacy of Health4Life in improving a further six pre-specified secondary outcomes of behavioural intentions, specifically, decreasing intentions to try smoking (tobacco cigarettes) and alcohol, and increasing intentions to be physically active, reduce screentime, meet sleep guidelines, and swap discretionary beverages for water.

## Methods

### Study design

A cRCT was conducted in 71 secondary schools across three Australian states from 2019 to 2022, with approval from the Human Research Ethics Committees of the University of Sydney (2018/882), Curtin University (HRE2019-0083), the University of Queensland (2,019,000,037), and relevant school sector ethics committees. This trial followed the Consolidated Standards of Reporting Trials (CONSORT) guidelines (completed CONSORT reporting checklist in Table S5) and was prospectively registered (ACTRN12619000431123).

### Procedure

A detailed description of power calculations, recruitment, randomization, and masking procedures has been provided elsewhere (Teesson et al., 2020). Briefly, power calculations utilized Heo and Leon’s method developed for longitudinal cRCTs (Heo & Leon, 2009), and the trial was powered for an anticipated retention rate of 70% by final follow-up. Schools were recruited through convenience and purposive sampling, with 519 schools (all under the jurisdiction of ethics boards that had granted approval) approached across NSW, QLD, and WA. Randomization was conducted by a biostatistician who was independent from recruitment using the Blockrand function in R, stratifying by site and school gender composition. As is standard for school-based cRCTs, allocation was not concealed from schools, participants, and researchers. All Year 7 students aged 11–13 years who attended the participating schools and were fluent in English were eligible. Participation required active student consent and either passive (opt-out) or active (opt-in) parental consent, depending on school ethics board requirements. Students completed surveys in class at baseline (July to October 2019), immediately post-intervention (September to December 2019), as well as 12 months (July to December 2020) and 24 months (July to December 2021) post-baseline. The intervention group was given access to the Health4Life intervention, while the active control group received the usual health education provided by their school.

### The Health4Life intervention

The Health4Life intervention is an eHealth program that educates students about the Big 6 lifestyle behaviours using a staged model of prevention that has universal and selective components. The universal components include six cartoon modules delivered during health education class, along with web-based tailored feedback, optional teacher-facilitated activities, and an optional companion smartphone app for use outside of class. Example slides from the cartoon modules are provided in Fig. S1. The program aims to provide evidence-based information, develop resistance and self-regulatory skills, modify perceptions of existing norms, and increase autonomous motivation. The accompanying app helps students track behaviours and set goals to encourage behaviour change. Students identified as “at risk” for two or more of the Big 6 at the 12- and/or 24-month survey occasion got access to selective intervention content covering cognitive behavioural and motivation enhancement techniques (Teesson et al., 2020). The intervention was co-designed with input from teachers, students, and experts. Detailed information about the intervention development and user testing is published elsewhere (Champion et al., 2020).

### Active control condition

The control schools provided their usual health education curriculum, and the teachers recorded the format and amount of any education related to the Big 6 in a logbook. Out of 35 control schools, 32 provided logbook data from 96 teachers. Most control school teachers (90 out of 96; 94%) reported teaching at least one lesson that covered one of the Big 6 health education topics during the first year of the trial.

### Outcomes

#### Behavioural intentions

At each timepoint, participants completed a self-report questionnaire that included six items assessing their intentions to engage in or change their health behaviours. Alcohol and physical activity items were measured with previously developed and tested measures (Newton et al., 2012; Sutherland et al., 2017). Items for sleep, discretionary beverages, screentime, and tobacco smoking were adapted from these measures. For alcohol and tobacco, participants reported how likely it was that they would try alcohol and tobacco (smoking) in the future on a scale of 0 (very unlikely) to 4 (very likely). For physical activity, screentime, discretionary beverages, and sleep, participants were presented with a 4-point scale ranging from 0 (not at all true of me) to 3 (very true of me) and asked—over the next 3 months—the extent to which they intended to: (a) be physically active on all or most days of the week; (b) reduce their screentime on all or most days of the week; (c) swap energy drinks, soft drinks, sports drinks, or cordial for water on all or most days of the week; and (d) sleep for [9–11 or 8–10] hours per night on all or most days of the week. The sleep question was adjusted to align with National Sleep Guidelines for the mean student age at time of survey completion (Department of Health, 2021).

### Statistical analyses

We used mixed effects ordinal logistic (and generalized for tobacco smoking intentions) regression models to investigate whether Health4Life improved behavioural intentions around the “Big 6”. We tested three different approaches (linear, quadratic, and categorical) for the timepoint variable for each behavioural intention outcome without any covariates. To account for the clustered longitudinal study design, we incorporated participants nested within schools as random effects. This approach accounts for the repeated measures from participants by incorporating random effects to account for the clustering of participants within schools and individuals over time. We used Akaike information criterion (AIC) and Bayesian information criterion (BIC) to identify the optimal approach to time for each outcome separately. Lower AIC and BIC values suggest better model fit, and published thresholds were used to determine the strength of evidence of fit differences to guide model selection (Fabozzi et al., 2014). If models were not easily distinguishable based on model fit values, we chose to use categorical time for consistency between outcomes and ease of parameter interpretation across outcomes. Analyses were conducted using the *lme4* (Bates et al., 2014) and *ordinal* (Christensen, 2022) packages in R (version 4.2.3).

After identifying the optimal time approach for each outcome, we tested the effect of the intervention on each outcome using a time-by-group interaction. The *α* level was set at a conservative 0·008 level based on a Bonferroni correction (*α* = 0·05/6) to account for testing of multiple outcomes. We controlled for sex at birth and school region (NSW metro, NSW regional, WA, and QLD) as both were used for stratified randomization of the cRCT. Intervention effects represent change in odds of moving up or down a response category on the intention scale (i.e. moving from 0 “not at all true of me” to 1). We conducted missing data analysis for each behavioural intention using ordinal logistic regression and examined differences in attrition between the intervention and control groups using binary logistic regression. Mixed effects models implemented using the *lme4* and *ordinal* packages handle missing data using a method known as maximum likelihood estimation, which takes advantage of available data to estimate the model parameters while accounting for the missingness in the data. We ran sensitivity analyses for each behavioural intention to investigate intervention effects in the subsamples who (1) were not meeting guidelines for each of the behaviours at baseline (i.e., those not meeting the 7 day/week MVPA guidelines, not having 9 to 11 h of sleep, exceeding 2 h of recreational screentime, reporting drinking sugar-sweetened beverages at least once per week), and (2) had not already tried alcohol and cigarettes at baseline. Table S4 provides summary statistics on sample sizes for each group.

## Results

**Fig. 1:**
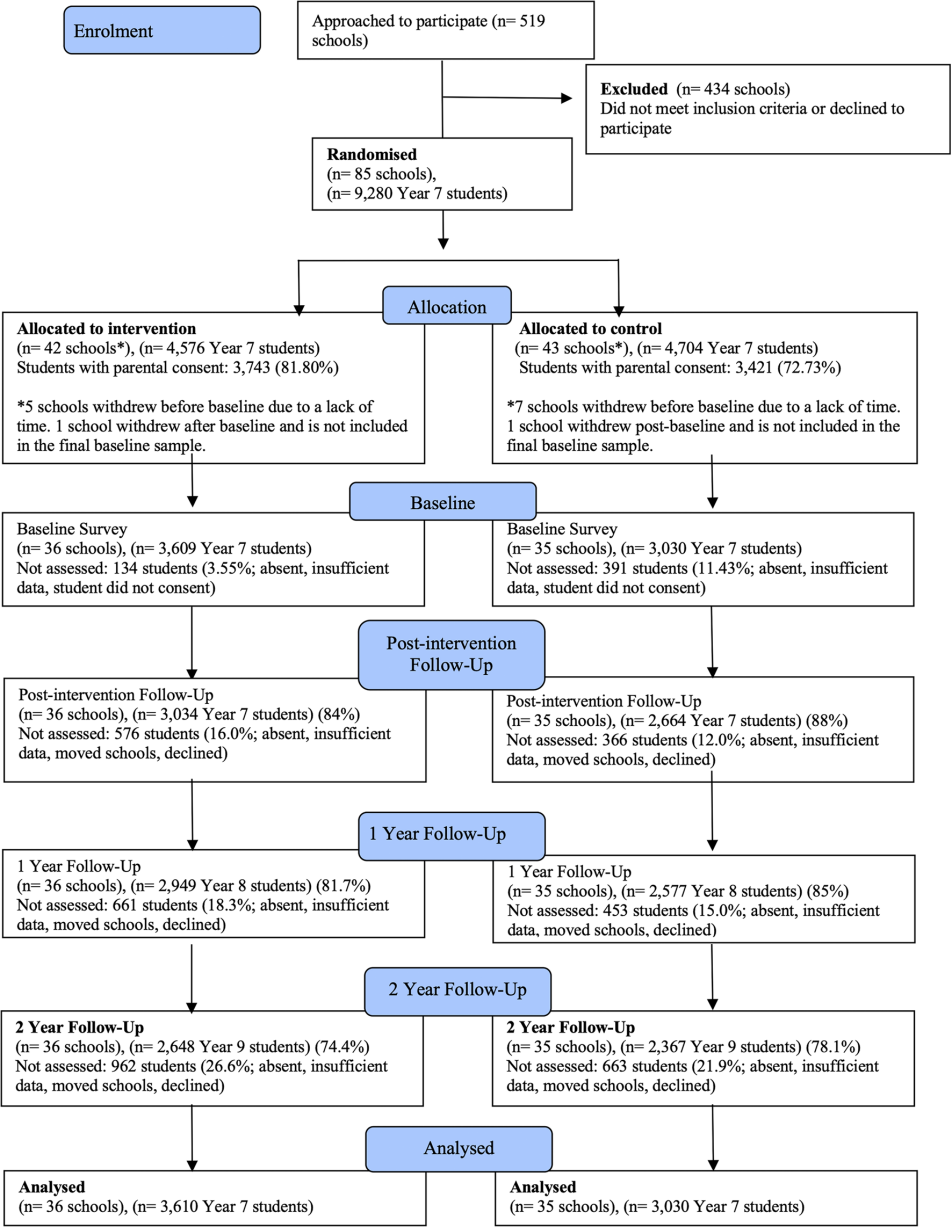
The CONSORT diagram for the Health4Life cluster randomized controlled trial

Detailed sample characteristics at baseline (*N* = 6639 students, 71 schools) are available in Table 1. Most students (*N* = 6454; 97.2%) completed at least one follow-up survey, and 5698 (85.8%) completed two or more follow-ups. Figure 1 contains the CONSORT diagram. Descriptive statistics for all behavioural intentions outcomes by intervention group are reported in Table 2. Results from the attrition analyses by demographic characteristics are reported elsewhere (Champion et al., 2023). No outcomes at baseline were differentially associated with attrition (Table S1). There was no evidence of a difference in the odds of attrition between the intervention and control group for any other outcome (Table S2).

**Table 1:** Characteristics of the sample at baseline

	Control	Intervention
*n*	3030	3609
Age	12.64 (0.51)	12.66 (0.50)
Birth sex (*N* (%))		
Male	1443 (47.8)	1859 (51.6)
Female	1532 (50.7)	1694 (47.0)
Prefer not to say	46 (1.5)	48 (1.3)
State (*N* (%))		
NSW	1495 (49.3)	2041 (56.6)
QLD	655 (21.6)	1133 (31.4)
WA	880 (29.0)	435 (12.1)
SES^a^ (*N* (%))		
Low	719 (26.7)	1033 (31.1)
Middle	1260 (46.8)	1407 (42.4)
High	713 (26.5)	882 (26.6)
School type (*N* (%))		
Independent	1893 (62.5)	1484 (41.1)
Catholic	269 (8.9)	990 (27.4)
Government	868 (28.6)	1135 (31.4)
School geographic area^b^ (*N* (%))		
Regional	380 (12.5)	306 (8.5)
Major city	2650 (87.5)	3303 (91.5)

^a^Family Affluence Scale (FAS III) scores were converted to ridit scores that compare socio-economic status with other people in the study sample, classified as lower (under 0.33), middle (0.33–0.66), or higher socio-economic status (0.66–1.0). ^b^Australian Statistical Geography Standard Remoteness Structure. Regional combines inner regional and outer regional

**Table 2:** Summary table of raw data for each outcome by time and intervention status

		Prevalence in each level (95% CI)			
		Baseline	Post-intervention	12 months	24 months
Alcohol use intentions (*N*, % (95% CI))					
Very unlikely	Control	679 (23.8 (22.3, 25.4))	570 (23.2 (21.6, 24.9))	482 (19.6 (18.1, 21.2))	395 (17.6 (16.1, 19.2))
	H4L	967 (28.1 (26.2, 29.6))	904 (32.1 (30.4, 33.8))	671 (24.2 (22.7, 25.9))	541 (22.4 (20.8, 24.1))
Unlikely	Control	394 (13.8 (12.6, 15.1))	293 (12.0 (10.8, 13.4))	266 (10.8 (9.6, 12.1))	192 (8.5 (7.5, 9.8))
	H4L	470 (13.6 (12.5, 14.8))	369 (13.1 (11.9, 14.4))	298 (10.8 (9.7, 12.0))	244 (10.1 (9.0, 11.4))
Unsure	Control	644 (22.6 (21.1, 24.2))	532 (21.6 (20.1, 23.3))	431 (17.5 (16.1, 19.1))	346 (15.4 (14.0, 16.9))
	H4L	732 (21.3 (19.9, 22.7))	525 (18.6 (17.2, 20.1))	466 (16.8 (15.5, 18.3))	345 (14.3 (13.0, 15.8))
Likely	Control	733 (25.7 (24.2, 27.4))	638 (25.69 (24.3, 27.7))	650 (26.4 (24.7, 28.2))	654 (29.1 (27.3, 31.0))
	H4L	835 (24.2 (22.8, 25.7))	648 (23.0 (21.5, 24.6))	726 (26.2 (24.6, 27.9))	646 (26.8 (25.1, 28.6))
Very likely	Control	399 (14.0( 12.8, 15.3))	423 (17.2 (15.8, 18.7))	631 (25.7 (24.0, 27.4))	660 (29.4 (27.5, 31.3))
	H4L	440 (12.8 (11.7, 13.9))	374 (13.3 (12.1, 14.6))	608 (22.0 (20.5, 23.5))	636 (26.4 (24.6, 28.2))
Tobacco (smoking) intentions (*N*, % (95% CI))					
Very unlikely	Control	2395 (84.4 (83.0, 85.7))	2012 (79.0 (77.4, 80.5))	1825 (74.2 (72.4, 75.8))	1600 (71.4 (69.5, 73.2))
	H4L	2854 (83.3 (82.0, 84.5))	2367 (84.1 (82.7, 82.7, 85.4))	2120 (76.7 (75.1, 78.3))	1742 (72.4 (70.6, 74.1))
Unlikely	Control	264 (9.3 (8.3, 10.4))	235 (12.8 (11.5, 14.1))	328 (13.3 (12.0, 14.7))	294 (13.1 (11.8, 14.6))
	H4L	328 (9.6 (8.6, 10.6))	229 (8.1 (7.2, 9.2))	298 (10.8 (9.7, 12.0))	276 (11.5 (10.3, 12.8))
Unsure	Control	116 (4.1 (3.4, 4.9))	129 (5.1 (4.3, 6.0))	152 (6.4 (5.5, 7.5))	177 (8.9 (6.9, 9.1))
	H4L	167 (4.9 (4.2, 5.6))	137 (4.9 (4.1, 5.7))	190 (6.9 (6.0, 7.9))	186 (7.7 (6.7, 8.9))
Likely	Control	40 (1.4 (1.0, 1.9))	32 (1.3 (0.9, 1.8))	70 (2.8 (2.3, 3.6))	82 (3.7 (2.9, 4.5))
	H4L	41 (1.2 (0.8, 1.6))	35 (1.2 (0.9, 1.7))	70 (2.5 (2.0, 3.2))	92 (3.8 (3.1, 4.7))
Very likely	Control	22 (0.8 (0.5, 1.2))	49 (1.9 (1.5, 2.5))	80 (3.3 (2.6, 4.0))	88 (3.9 (3.2, 4.8))
	H4L	36 (1.1 (0.8, 1.5))	47 (1.7 (1.3, 2.2))	85 (3.1 (2.5, 3.8))	111 (4.6 (3.8, 5.5))
MVPA intentions (*N*,% (95% CI))					
Not at all true of me	Control	122 (4.3 (3.6, 5.1))	122 (5.0 (4.2, 5.9))	145 (5.69 (5.1, 6.9))	131 (5.6 (5.0, 6.9))
	H4L	158 (4.6 (4.0, 5.4))	1506 (5.3 (4.6, 6.2))	161 (5.8 (5.0, 6.8))	167 (6.9 (6.0, 8.0))
Not very true of me	Control	246 (8.7 (7.7, 9.8))	201 (8.2 (7.1, 9.3))	190 (7.8 (6.7, 8.9))	208 (9.3 (8.2, 10.6))
	H4L	317 (9.2 (8.3, 10.3))	216 (7.7 (6.7, 8.7))	253 (9.2 (8.2, 10.3))	236 (9.8 (8.7, 11.1))
Somewhat true of me	Control	975 (34.3 (32.6, 36.1))	814 (33.0 (31.2, 34.9))	816 (33.3 (31.5, 35.2))	736 (32.9 (30.9, 34.8))
	H4L	1224 (35.7 (34.1, 37.3))	915 (32.5 (30.7, 24.2))	944 (34.2 (32.5, 36.0))	764 (31.8 (29.9, 33.7))
Very true of me	Control	1496 (52.7 (5039, 54.5))	1326 (53.8 (51.9, 55.8))	1299 (53.0 (51.0, 55.0))	1164 (52.0 (49.9, 54.1))
	H4L	1731 (50.5 (48.8, 52.1))	1538 (24.6 (52.7, 56.4))	1399 (50.7 (48.9, 52.6))	1238 (51.5 (49.5, 53.5))
Screentime intentions (*N*, % (95% CI))					
Not at all true of me	Control	412 (14.5 (14.9, 17.3))	317 (12.9 (11.6, 14.3))	485 (19.9 (18.3, 21.5))	442 (19.8 (18.2, 21.5))
	H4L	549 (16.1 (14.9, 17.3))	322 (11.4 (10.3, 12.7))	509 (18.6 (17.2, 20.1))	476 (19.8 (18.3, 21.5))
Not very true of me	Control	813 (28.7 (27.1, 30.4))	674 (27.5 (25.7, 29.3))	782 (32.0 (30.2, 33.9))	758 (33.9 (32.0, 35.9))
	H4L	946 (27.7 (26.2, 29.2))	669 (23.8 (22.2, 25.4))	812 (29.7 (28.0, 31.4))	750 (31.2 (29.4, 33.1))
Somewhat true of me	Control	1149 (40.6 (38.8, 42.4))	1009 (41.1 (39.2, 43.1))	848 (34.7 (32.9, 36.6))	744 (33.3 (31.4, 35.3))
	H4L	1400 (41.0 (39.3, 42.6))	1181 (42.0 (10.2, 43.8))	987 (36.1 (34.3, 37.9))	780 (32.5 (30.6, 34.4))
Very true of me	Control	459 (16.2 (14.9, 17.6))	454 (18.5 (17.0, 20.1))	327 (13.4 (12.1, 17.8))	292 (13.1 (11.7, 14.5))
	H4L	521 (15.3 (14.1, 16.5))	642 (22.8 (21.3, 24.4))	428 (15.6 (14.3, 17.1))	395 (16.5 (15.0, 18.0))
Sleep intentions (*N*, % (95% CI))					
Not at all true of me	Control	398 (14.1 (12.8, 15.4))	275 (11.1 (10.0, 12.4))	333 (13.6 (12.3, 15.0))	264 (11.8 (10.6, 13.2))
	H4L	451 (13.2 (12.1, 14.4))	238 (8.5 (7.5, 9.6))	298 (10.9 (9.4, 12.1))	267 (11.1 (9.9, 12.4))
Not very true of me	Control	641 (22.7 (21.1, 24.2))	513 (20.8 (19.2, 22.4))	484 (19.8 (18.2, 21.4))	402 (18.0 (16.5, 19.7))
	H4L	723 (21.2 (19.8, 22.6))	454 (16.1 (14.8, 17.6))	487 (17.7 (16.4, 19.2))	414 (17.2 (15.8, 18.8))
Somewhat true of me	Control	982 (34.7 (33.0, 36.5))	905 (37.1 (35.2, 39.0))	893 (36.5 (34.6, 38.4))	802 (35.9 (34.0, 38.0))
	H4L	1222 (35.8 (34.2, 37.4))	1005 (35.7 (34.0, 37.5))	1034 (37.7 (35.9, 39.5))	852 (35.5 (33.6, 37.4))
Very true of me	Control	808 (28.6 (26.9, 30.3))	765 (31.0 (29.2, 32.8))	739 (30.2 (28.4, 32.0))	763 (34.2 (32.2, 36.2))
	H4L	1022 (29.9 (28.4, 31.5))	1115 (39.7 (37.9, 41.5))	926 (33.7 (32.0, 25.5))	870 (36.2 (34.3, 38.1))
Discretionary beverage intentions (*N*, % (95% CI))					
Not at all true of me	Control	583 (20.6 (19.1, 22.1))	412 (16.7 (15.3, 18.3))	450 (18.4 (16.9, 20.0))	371 (16.6 (15.1, 18.2))
	H4L	670 (19.5 (18.3, 20.9))	439 (15.6 (13.3, 15.9))	485 (17.6 (16.2, 19.1))	362 (16.1 (13.7, 16.6))
Not very true of me	Control	419 (17.8 (13.5, 16.1))	351 (14.3 (12.9, 15.7))	415 (17.0 (15.5, 18.5))	345 (15.4 (14.0, 17.0))
	H4L	614 (17.9 (16.7, 19.2))	409 (14.5 (13.3, 15.9))	465 (16.9 (15.5, 18.3))	354 (14.8 (13.4, 16.2))
Somewhat true of me	Control	840 (29.7 (287.0, 31.4))	672 (27.3 (25.6, 29.1))	662 (27.0 (25.3, 28.8))	597 (26.7 (24.9, 28.6))
	H4L	1051 (30.7 (29.1, 32.2))	820 (29.2 (27.5, 30.9))	766 (27.8 (26.2, 29.5))	656 (27.4 (25.6, 29.2))
Very true of me	Control	991 (35.0 (33.2, 36.8))	1026 (41.7 (39.8, 43.6))	921 (37.6 (35.7, 39.6))	921 (41.2 (39.2, 43.3))
	H4L	1093 (31.9 (30.3, 33.5))	1144 (40.7 (38.9, 42.25))	1040 (37.7 (35.9, 39.6))	1026 (42.8 (40.8, 44.8))

*H4L* Health4Life intervention group, *CI* confidence interval, *SD* standard deviation, *MVPA* moderate-vigorous physical activity

### Intervention effects

**Fig. 2:**
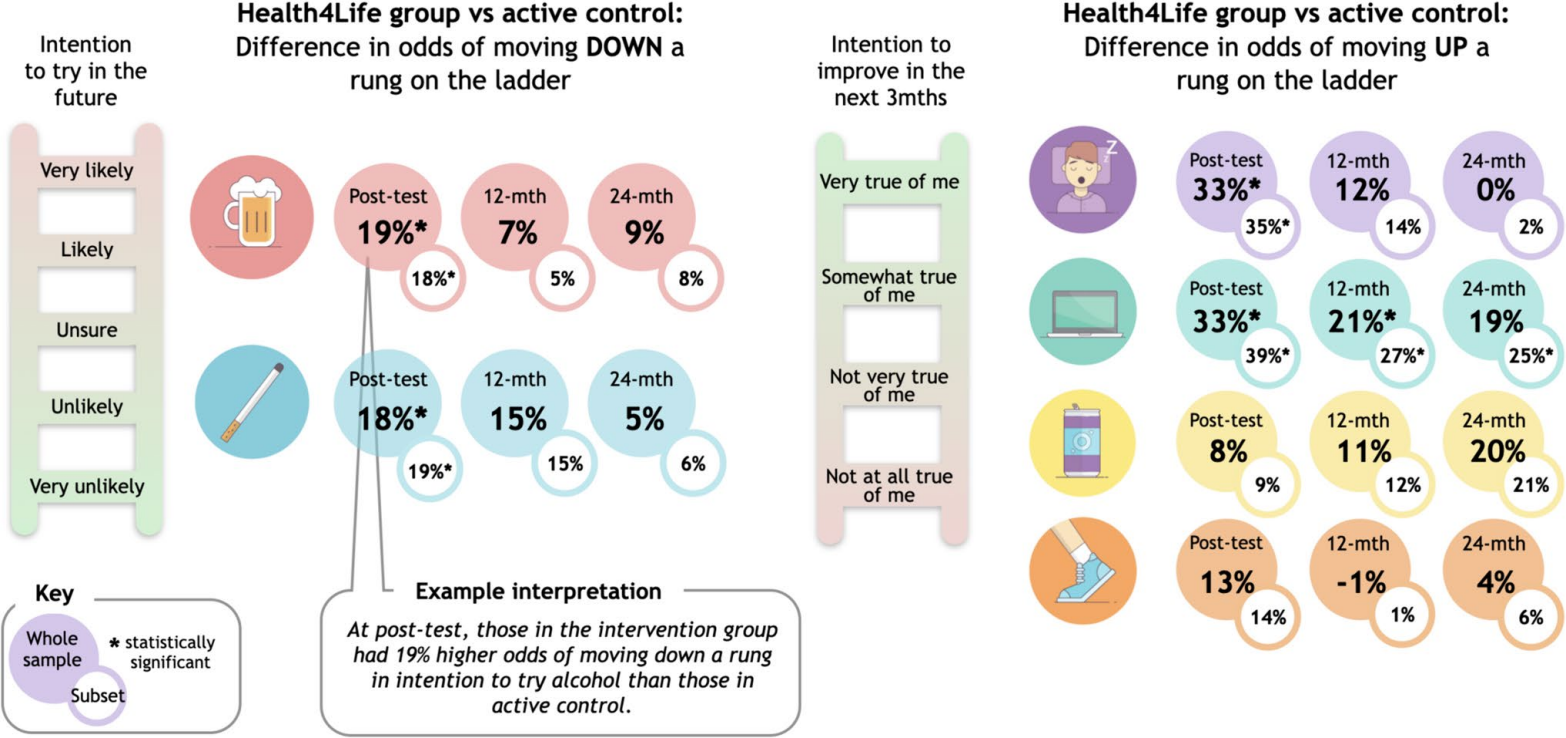
Visual summary illustrating the difference between the intervention group and control group in odds of shifting intentions

Model fit statistics for the best fitting unconditional growth models and specification of time are reported in Table S3. Group-by-time interaction parameters detailing the intervention effects on behavioural intentions are presented graphically in Fig. 2 and are summarized in Table 3. Compared to the control groups, students in the intervention group had significantly lower odds (or rates for tobacco) of changing to a higher intention to try alcohol or tobacco (smoking) in the future from baseline to post-intervention (alcohol *OR* = 0.81, 95% *CI* = 0.71, 0.93, *p* = 0.002; tobacco *IRR* = 0.82, 95% *CI* = 0.71, 0.94, *p* = 0.005) (Fig. 2). However, effects between intervention and control groups were no longer significant at 12 or 24 months relative to baseline. For sleep, those in the intervention group had higher odds of changing to higher intentions to get the recommended amount of sleep from baseline to post-intervention follow-up (*OR* = 1.33, 95% *CI* = 1.16, 1.52, *p* < 0.001); however, effects were again no longer significant at 12 or 24 months. For screentime, those in the intervention group had higher odds of changing to higher intentions to reduce screentime, both at post-intervention follow-up (*OR* = 1.33, 95% *CI* = 1.16, 1.51, *p* < 0.001) and 12-month follow-up (*OR* = 1.21, 95% *CI* = 1.06, 1.39, *p* = 0.005), relative to baseline. However, there was little evidence for a significant effect at 24 months. There was also little evidence for effects of the intervention on the odds of having higher intentions to improve MVPA and discretionary drink consumption at any follow-up timepoint relative to baseline (see Fig. 2 and Table 3).

**Table 3:** Summary table of mixed effects ordinal logistic regressions and linear mixed effects regression investigating the effect of the intervention on odds of being in higher intentions levels

	*Log odds*	*Std. error*	*OR (95% CI)*	*p*
Alcohol use intentions				
Post-intervention	− 0.207	0.066	0.81 (0.71, 0.93)	.002
12 months	− 0.067	0.067	0.93 (0.82, 1.07)	.313
24 months	− 0.098	0.069	0.91 (0.79, 1.04)	.158
Tobacco (smoking) intentions^a^				
Post-intervention	− 0.204	0.073	0.82 (0.71, 0.94)	.005
12 months	− 0.157	0.067	0.85 (0.75, 0.98)	.019
24 months	− 0.056	0.067	0.95 (0.83, 1.08)	.406
MVPA intentions				
Post-intervention	0.118	0.072	1.13 (0.98, 1.30)	.100
12 months	− 0.007	0.072	0.99 (0.86, 1.14)	.928
24 months	0.043	0.074	1.04 (0.90, 1.21)	.567
Screentime intentions				
Post-intervention	0.282	0.068	1.325 (1.16, 1.51)	< .001
12 months	0.193	0.069	1.213 (1.06, 1.39)	.005
24 months	0.178	0.071	1.195 (0.04, 1.37)	.012
Sleep intentions				
Post-intervention	0.284	0.068	1.33 (1.16, 1.52)	< .001
12 months	0.112	0.068	1.12 (0.98, 1.28)	.101
24 months	0.000	0.071	1.00 (0.87, 1.15)	.997
Discretionary beverage intentions				
Post-intervention	0.081	0.068	1.08 (0.95, 1.24)	.235
12 months	0.108	0.068	1.11 (0.97, 1.27)	.114
24 months	0.179	0.071	1.20 (1.04, 1.37)	.011

Significance value = 0.007; *CI* confidence interval. ^a^Model is mixed effects negative binomial regression to account for zero-inflated data with overdispersion. Effect size is rate ratio

When intervention effects on behavioural intentions were tested in the subset of students who were not meeting recommended health guidelines for the Big 6 at baseline, results largely followed similar patterns to the primary analyses (Fig. 2 and Table S4). However, intervention effects on screentime intentions remained significant at 24-month follow-up among those who were exceeding the 2-h daily sedentary recreational screentime at baseline.

### Intervention fidelity and active control

Of the 3157 students in the intervention group with available lesson completion data, 62.1% (1960) completed all six lessons. Of all intervention students, 11.3% (407) accessed the optional universal Health4Life app content and 0.1% (5) accessed the selective booster content. A dose–response analysis is available in the main outcomes study (Champion et al., 2023) but the present study does not differentiate by level of engagement. As is to be expected due to the curriculum requiring education around lifestyle behaviours, most control teachers at control schools (*N* = 90; 94%) reported delivering one or more lessons covering at least one of the Big 6.

## Discussion

This study reports findings from a cRCT on the efficacy of the MHBC Health4Life intervention on improving behavioural intentions around six key health behaviours. At post-intervention, adolescents in the Health4Life group had significantly improved intentions to reduce their screentime, achieve the recommended sleep duration, and not try alcohol and tobacco (smoking) in the future. These effects persisted up to 12 months post-intervention for screentime, however did not persist for sleep, alcohol, or tobacco use intentions. There was little evidence to suggest that the intervention was more efficacious than an active control in improving intentions to engage in physical activity on all or most days of the week or to reduce discretionary beverage consumption.

A core benefit of MHBC interventions is their ability to target several health behaviours efficiently, capitalizing on behavioural clustering and evidence that one health behaviour change is associated with changes in others (Prochaska et al., 2008). Research has not explicitly explored behavioural *intention* clustering. However, the present study demonstrates that one 6-week MHBC eHealth intervention changed intentions for four of six target behaviours among early adolescents, immediately post-intervention. Nevertheless, it is important to investigate possible explanations for the absence of effects on intentions related to physical activity and to discretionary beverage consumption; and how to sustain effects in the longer term.

Although not measured in the Health4Life study, some psychological factors known to influence the development of behavioural intentions include anticipated pride, anticipated regret, cognitive attitudes (e.g. perceived ease and importance of behaviour), and affective attitudes (e.g. perceived pleasantness of behaviour) (Stevens et al., 2019). The discretionary beverage questionnaire wording itself may have been too limited for participants to identify with anticipated pride or regret; i.e., young people may not anticipate regret due to consuming these beverages twice a week, or conversely may not feel pride in the notion of abstaining from discretionary beverages on “most or all days,” as specified in the question. Of note, intervention effects on discretionary beverage intentions were the only effects to increase in magnitude over the 24 months (albeit not reaching statistical significance after Bonferroni correction), with the intervention group 20% more likely than controls to have improved intentions at 24 months. By contrast, for physical activity, intentions were already high in both groups at baseline, with over 85% of participants saying that it was somewhat or very true that they intend to be physically active on “most or all” days. Thus, there is a potential ceiling effect in the measurement of physical activity intentions.

Although there were post-intervention effects on sleep, alcohol, and tobacco use intentions, it is important to interrogate why these effects were not sustained in the longer term, as intention temporal stability (i.e. the durability of intentions over time) strengthens the predictive power of intentions on behaviour change (Conner & Norman, 2022). It is possible that intention temporal stability would have been improved if app usage had been higher, as the app was intended to be used for the full 24-month period following the initial in-class program and included self-monitoring and goal-setting content, which likely would maintain intentionality. Moreover, the 12- and 24-month follow-up occurred during periods of restrictions and/or lockdowns for COVID-19 infection control, which possibly impacted the durability of intentions. Finally, the lack of temporal stability around intentions not to try alcohol “in the future” (as specified by the questionnaire item) may be due to students nearing the legal age of alcohol consumption throughout the trial period. The intervention did not advocate for lifetime alcohol abstinence, but rather to delay onset of drinking and harmful drinking; thus, it is expected that as students progress through adolescence they may intend to try alcohol at some point in their future. It is possible that the intervention changed students’ intentions to try alcohol prior to the age of 18 (the legal drinking age in Australia); however, this was not captured by the questionnaire.

### Strengths and limitations

This study has several notable strengths. First, it utilizes a large, multi-state sample, increasing the statistical power and generalizability of the findings. Second, the study employs a longitudinal design, spanning two years and consisting of four timepoints, allowing for the examination of changes and patterns in behavioural intentions over an extended period. However, these findings should be considered in light of a number of limitations. The measures used to identify intentions around sleep, screentime, discretionary beverage consumption, and smoking were purpose-built for this study and not externally validated, potentially making the findings vulnerable to measurement and reporting bias. However, the physical activity and alcohol measures have been used in previous prevention research (Newton et al., 2012; Sutherland et al., 2017), and formed the basis of adaptation for the aforementioned measures. Further, the intervention content focused on sugar-sweetened beverages, but the questionnaire item for dietary intentions did not limit to sugar-sweetened beverages and exclude their “diet”/sugar-free counterparts. The study was robustly powered for the primary outcome analyses, but a priori power analyses were not done for secondary behavioural intentions. However, the sample size is substantial and is likely well powered to detect effects on these six outcomes. Moreover, the reliance on self-reported measures for assessing behavioural intentions can be subject to various biases, including memory recall biases, social desirability bias, and response bias, which could affect the accuracy and reliability of the reported intentions. In the context of behaviour change interventions, participants may feel pressure to report intentions to change their behaviour in a more favourable light, leading to an overestimation of the intervention’s effectiveness. However, the study used assurances of confidentiality to reduce the likelihood of participants responding in a socially desirable way.

Further limitations are the unknown impacts of the COVID-19 pandemic on outcomes, as well as the absence of data on intentions to try e-cigarettes or “vaping”, despite high prevalence among adolescents and evidence that vaping generates a three-fold increased risk of combustible cigarette smoking initiation (Baenziger et al., 2021). Research indicates that individual-based eHealth intervention could inadvertently increase health inequities by favouring individuals with greater access to digital resources and higher levels of digital literacy (Honeyman et al., 2020). However, the Health4Life intervention was universal, as such it was delivered to all students at included schools and was offered to all students at control schools following trial completion. Moreover, the core content of the intervention was delivered in class, thereby not differentiating by student’s home context. Finally, the intervention relies on theories of behaviour change that focus on individual motivation, and therefore it does not consider environmental factors that likely also influence behaviour in these domains.

### Opportunities for future research

Given that the Health4Life intervention was effective in improving intentions around alcohol use, tobacco (smoking), sleep duration and screentime, yet behaviour change did not follow, further research should interrogate this intention-behaviour gap. Evidence shows that intentions that are more stable over time are stronger behavioural predictors (Conner & Norman, 2022), so future research should aim to sustain intervention effects on intentions at 12 and 24 months, for example through increasing adherence to the app or other booster intervention components, like “implementation intention” activities (Adriaanse et al., 2011; Bélanger-Gravel et al., 2013; Malaguti et al., 2020). Or as an alternative to the app, additional content incorporating goal-setting and self-monitoring could be incorporated into the main intervention. Finally, in light of evidence that vaping predicts increased uptake of tobacco smoking, future research should examine intentions to use e-cigarettes (Baenziger et al., 2021).

## Conclusion

The Health4Life MHBC intervention was effective in improving behavioural intentions for alcohol use, tobacco smoking, sleep duration, and screentime in the short term. However, most of these effects were not sustained in the longer term. The low uptake of the intervention’s app-based components may have contributed to these findings. Disruptions to participants’ lives due to the COVID-19 pandemic and related restrictions may also have played a role. Future research should focus on sustaining intention effects over time to minimize the intention-behaviour gap and incorporating supportive environments for individuals to trial new behaviours. By addressing these areas, MHBC interventions may be optimized to effectively sustain behavioural intentions, ultimately increasing the likelihood of behaviour change.

## Contributions to knowledge

What does this study add to existing knowledge?

- To date, no studies have examined whether a single MHBC intervention can impact intentions to change behaviours across physical activity, diet, sleep, sedentary recreational screentime, alcohol use, and tobacco smoking.
- This study demonstrates that a school-based eHealth MHBC intervention can improve adolescents’ intentions regarding alcohol, tobacco, sleep, and screentime. However, it highlights the challenge of sustaining intention changes over time, suggesting a need for strategies to bridge the gap between intentions and actual behaviour.

What are the key implications for public health interventions, practice, or policy?

- This study provides some preliminary evidence for the development and integration of universal eHealth MHBC interventions into school-based programs, particularly if combined with strategies to promote long-term behaviour change. The findings urge the exploration of additional intervention components that can help translate initial intention shifts into sustained healthy habits.

## Supplementary Information

Below is the link to the electronic supplementary material.

Supplementary file1 (DOCX 418 KB)

## Data Availability

De-identified participant data will be made available to researchers on reasonable request to K.E.C. (katrina.champion@sydney.edu.au) when accompanied by study protocol and analysis plan. Data will be shared after the approval of a proposal by a committee of the current research team with a signed data access agreement.

## Availability of code

The statistical analysis code (syntax) will be made available to researchers on reasonable request to SO (siobhan.odean@sydney.edu.au).

## References

[CR1] Adriaanse, M. A., Vinkers, C. D. W., De Ridder, D. T. D., Hox, J. J., & De Wit, J. B. F. (2011). Do implementation intentions help to eat a healthy diet? A systematic review and meta-analysis of the empirical evidence. *Appetite,**56*(1), 183–193. 10.1016/j.appet.2010.10.012 21056605 10.1016/j.appet.2010.10.012

[CR2] Baenziger, O. N., Ford, L., Yazidjoglou, A., Joshy, G., & Banks, E. (2021). E-cigarette use and combustible tobacco cigarette smoking uptake among non-smokers, including relapse in former smokers: Umbrella review, systematic review and meta-analysis. *British Medical Journal Open,**11*(3), e045603. 10.1136/bmjopen-2020-045603 10.1136/bmjopen-2020-045603 PMC8011717 33785493

[CR3] Ball, J., Grucza, R., Livingston, M., ter Bogt, T., Currie, C., & de Looze, M. (2023). The great decline in adolescent risk behaviours: Unitary trend, separate trends, or cascade? *Social Science & Medicine,**317*, 115616. 10.1016/j.socscimed.2022.115616 36563586 10.1016/j.socscimed.2022.115616

[CR4] Bates, D., Mächler, M., Bolker, B., & Walker, S. (2014). Fitting linear mixed-effects models using lme4. *arXiv Preprint *arXiv:1406.5823.

[CR5] Bélanger-Gravel, A., Godin, G., & Amireault, S. (2013). A meta-analytic review of the effect of implementation intentions on physical activity. *Health Psychology Review,**7*(1), 23–54. 10.1080/17437199.2011.560095

[CR6] Champion, K. E., Gardner, L. A., McGowan, C., Chapman, C., Thornton, L., Parmenter, B., McBride, N., Lubans, D. R., McCann, K., Spring, B., Teesson, M., & Newton, N. C. (2020). A web-based intervention to prevent multiple chronic disease risk factors among adolescents: Co-design and user testing of the Health4Life school-based program. *JMIR Form Res,**4*(7), e19485. 10.2196/19485 32720898 10.2196/19485 PMC7420628

[CR7] Champion, K. E., Chapman, C., Gardner, L. A., Sunderland, M., Newton, N. C., Smout, S., Thornton, L. K., Hides, L., McBride, N., Allsop, S. J., Mills, K., Kay-Lambkin, F., Teesson, M., Slade, T., & the Health4Life team. (2021). Lifestyle risks for chronic disease among Australian adolescents: A cross-sectional survey. *Medical Journal of Australia,**216*(3), 156–157. https://www.mja.com.au/journal/2022/216/3/lifestyle-risks-chronic-disease-among-australian-adolescents-cross-sectional. 10.5694/mja2.51333 34747039

[CR8] Champion, K. E., Newton, N. C., Gardner, L. A., Chapman, C., Thornton, L., Slade, T., Sunderland, M., Hides, L., McBride, N., O’Dean, S., Kay-Lambkin, F., Allsop, S., Lubans, D. R., Parmenter, B., Mills, K., Spring, B., Osman, B., Ellem, R., Smout, S., Whife, J., Stewart, C., McCann, K. M., Catakovic, A., Hunter, E., & Teesson, M., on behalf of the Health4Life Team. (2023). Health4Life eHealth intervention to modify multiple lifestyle risk behaviours among adolescent students in Australia: A cluster-randomised controlled trial. *The Lancet Digital Health*, *5*(5), e276–e287. 10.1016/S2589-7500(23)00028-6 10.1016/S2589-7500(23)00028-6 37032200

[CR9] Christensen, R. H. B. (2022). *Regression models for ordinal data [R package ordinal version 2022.11–16]*. Retrieved from: https://CRAN.R-project.org/package=ordinal

[CR10] Conner, M., & Norman, P. (2022). Understanding the intention-behavior gap: The role of intention strength. *Frontiers in Psychology,**13*, 923464. 10.3389/fpsyg.2022.923464 35992469 10.3389/fpsyg.2022.923464 PMC9386038

[CR11] Cooke, R., Dahdah, M., Norman, P., & French, D. P. (2016). How well does the theory of planned behaviour predict alcohol consumption? A systematic review and meta-analysis. *Health Psychology Review,**10*(2), 148–167. 10.1080/17437199.2014.947547 25089611 10.1080/17437199.2014.947547 PMC4867851

[CR12] Department of Health. (2021). *Physical activity and exercise guidelines for all Australians: For children and young people (5 to 17 years)*. Retrieved from: https://www.health.gov.au/topics/physical-activity-and-exercise/physical-activity-and-exercise-guidelines-for-all-australians/for-children-and-young-people-5-to-17-years

[CR13] Due, P., Krølner, R., Rasmussen, M., Andersen, A., Trab Damsgaard, M., Graham, H., & Holstein, B. E. (2011). Pathways and mechanisms in adolescence contribute to adult health inequalities. *Scandinavian Journal of Public Health,**39*(6_suppl), 62–78. 21382850 10.1177/1403494810395989

[CR14] Fabozzi, F. J., Focardi, S. M., Rachev, S. T., Arshanapalli, B. G., & Hoechstoetter, M. (2014). *The basics of financial econometrics: Tools, concepts, and asset management applications*. John Wiley & Sons.

[CR15] Gariepy, G., Danna, S., Gobiņa, I., Rasmussen, M., Gaspar de Matos, M., Tynjälä, J., Janssen, I., Kalman, M., Villeruša, A., Husarova, D., Brooks, F., Elgar, F. J., Klavina-Makrecka, S., Šmigelskas, K., Gaspar, T., & Schnohr, C. (2020). How are adolescents sleeping? Adolescent sleep patterns and sociodemographic differences in 24 European and North American countries. *Journal of Adolescent Health,**66*(6), S81–S88. 10.1016/j.jadohealth.2020.03.013 10.1016/j.jadohealth.2020.03.013 32446613

[CR16] GBD 2019 Diseases and Injuries Collaborators. (2019). *Non-communicable diseases—Level 1 cause*. Global Burden of Disease: The Lancet GBD Cause and Risk Summaries. https://www.thelancet.com/pb-assets/Lancet/gbd/summaries/diseases/non-communicable-diseases.pdf

[CR17] Heo, M., & Leon, A. C. (2009). Sample size requirements to detect an intervention by time interaction in longitudinal cluster randomized clinical trials. *Statistics in Medicine,**28*(6), 1017–1027. 10.1002/sim.3527 19153969 10.1002/sim.3527 PMC2758777

[CR18] Honeyman, M., Maguire, D., Evans, H., & Davies, A. (2020). *Digital technology and health inequalities: A scoping review*. Public Health Wales NHS Trust.

[CR19] Keller, A., Torre, B. D., & S. (2015). Sugar-sweetened beverages and obesity among children and adolescents: A review of systematic literature reviews. *Childhood Obesity,**11*(4), 338–346. 10.1089/chi.2014.0117 26258560 10.1089/chi.2014.0117 PMC4529053

[CR20] Malaguti, A., Ciocanel, O., Sani, F., Dillon, J. F., Eriksen, A., & Power, K. (2020). Effectiveness of the use of implementation intentions on reduction of substance use: A meta-analysis. *Drug and Alcohol Dependence,**214*, 108120. 10.1016/j.drugalcdep.2020.108120 32622228 10.1016/j.drugalcdep.2020.108120

[CR21] Murray, C. J. L., Aravkin, A. Y., Zheng, P., Abbafati, C., Abbas, K. M., Abbasi-Kangevari, M., Abd-Allah, F., Abdelalim, A., Abdollahi, M., Abdollahpour, I., Abegaz, K. H., Abolhassani, H., Aboyans, V., Abreu, L. G., Abrigo, M. R. M., Abualhasan, A., Abu-Raddad, L. J., Abushouk, A. I., Adabi, M., et al. (2020). Global burden of 87 risk factors in 204 countries and territories, 1990–2019: A systematic analysis for the Global Burden of Disease Study 2019. *The Lancet*, *396*(10258), 1223–1249. 10.1016/S0140-6736(20)30752-2 10.1016/S0140-6736(20)30752-2 PMC7566194 33069327

[CR22] Newton, N. C., Teesson, M., Barrett, E. L., Slade, T., & Conrod, P. J. (2012). The CAP study, evaluation of integrated universal and selective prevention strategies for youth alcohol misuse: Study protocol of a cluster randomized controlled trial. *BMC Psychiatry,**12*(1), 118. 10.1186/1471-244X-12-118 22906138 10.1186/1471-244X-12-118 PMC3502100

[CR23] O’Dean, S., Sunderland, M., Newton, N. C., Gardner, L. A., Teesson, M., Chapman, C., Thornton, L., Slade, T., Hides, L., McBride, N., Kay-Lambkin, F., Allsop, S., Lubans, D. R., Parmenter, B., Mills, K. L., Spring, B. J., Osman, B., Ellem, R., Smout, S., McCann, K., Hunter, E., Catakovic, A., & Champion, K., The Health4Life Team. (2023). Effects of an eHealth intervention to modify multiple lifestyle risk behaviours among adolescents: Secondary outcomes from the Health4Life cluster-randomised controlled trial. *Medical Journal of Australia*, *220*(8), 417–424. 10.5694/mja2.52279 10.5694/mja2.52279 38613175

[CR24] Patton, G. C., Sawyer, S. M., Santelli, J. S., Ross, D. A., Afifi, R., Allen, N. B., Arora, M., Azzopardi, P., Baldwin, W., & Bonell, C. (2016). Our future: A Lancet commission on adolescent health and wellbeing. *The Lancet,**387*(10036), 2423–2478. 10.1016/S0140-6736(16)00579-1 PMC5832967 27174304

[CR25] Plotnikoff, R. C., Costigan, S. A., Karunamuni, N., & Lubans, D. R. (2013). Social cognitive theories used to explain physical activity behavior in adolescents: A systematic review and meta-analysis. *Preventive Medicine,**56*(5), 245–253. 10.1016/j.ypmed.2013.01.013 23370047 10.1016/j.ypmed.2013.01.013

[CR26] Prochaska, J. J., Spring, B., & Nigg, C. R. (2008). Multiple health behavior change research: An introduction and overview. *Preventive Medicine,**46*(3), 181–188. 18319098 10.1016/j.ypmed.2008.02.001 PMC2288583

[CR27] Riebl, S. K., Estabrooks, P. A., Dunsmore, J. C., Savla, J., Frisard, M. I., Dietrich, A. M., Peng, Y., Zhang, X., & Davy, B. M. (2015). A systematic literature review and meta-analysis: The Theory of Planned Behavior’s application to understand and predict nutrition-related behaviors in youth. *Eating Behaviors,**18*, 160–178. 10.1016/j.eatbeh.2015.05.016 26112228 10.1016/j.eatbeh.2015.05.016

[CR28] Ryan, R. M., & Deci, E. L. (2000). Intrinsic and extrinsic motivations: Classic definitions and new directions. *Contemporary Educational Psychology,**25*(1), 54–67. 10.1006/ceps.1999.1020 10620381 10.1006/ceps.1999.1020

[CR29] Singh, B., Ahmed, M., Staiano, A. E., Gough, C., Petersen, J., Vandelanotte, C., Kracht, C., Huong, C., Yin, Z., & Vasiloglou, M. F. (2024). A systematic umbrella review and meta-meta-analysis of eHealth and mHealth interventions for improving lifestyle behaviours. *Npj Digital Medicine,**7*(1), 179. 38969775 10.1038/s41746-024-01172-y PMC11226451

[CR30] Smout, S., Champion, K., O’Dean, S., Teesson, M., Gardner, L., & Newton, N. (2024). Anxiety, depression and distress outcomes from the Health4Life intervention for adolescent mental health: A cluster-randomized controlled trial. *Nature Mental Health*, 1–10. 10.1038/s44220-024-00246-w

[CR31] Stevens, C. J., Gillman, A. S., Gardiner, C. K., Montanaro, E. A., Bryan, A. D., & Conner, M. (2019). Feel good now or regret it later? The respective roles of affective attitudes and anticipated affective reactions for explaining health-promoting and health risk behavioral intentions. *Journal of Applied Social Psychology,**49*(6), 331–348. 10.1111/jasp.12584 31511748 10.1111/jasp.12584 PMC6738954

[CR32] Sutherland, R., Wiggers, J., Campbell, E., Lubans, D. R., Morgan, P. J., Nathan, N., Wolfenden, L., Okely, A. D., Gillham, K., Oldmeadow, C., Reeves, P., Williams, A., & Davies, L. (2017). *Physical Activity 4 Everyone: Outcomes of a multi-component schoolbased physical activity intervention for adolescents.* NSW Ministry of Health. Retrieved from: https://www.health.nsw.gov.au/research/Publications/physical-activity-for-everyone.pdf

[CR33] Taylor, D., Bury, M., Campling, N., Carter, S., Garfied, S., Newbould, J., & Rennie, T. (2006). *A review of the use of the Health Belief Model (HBM), the Theory of Reasoned Action (TRA), the Theory of Planned Behaviour (TPB) and the Trans-Theoretical Model (TTM) to study and predict health related behaviour change* (pp. 1–215). National Institute for Health and Clinical Excellence.

[CR34] Teesson, M., Champion, K. E., Newton, N. C., Kay-Lambkin, F., Chapman, C., Thornton, L., Slade, T., Sunderland, M., Mills, K., & Gardner, L. A. (2020). Study protocol of the Health4Life initiative: A cluster randomised controlled trial of an eHealth school-based program targeting multiple lifestyle risk behaviours among young Australians. *British Medical Journal Open,**10*(7), e035662. 10.1136/bmjopen-2019-035662 PMC7359380 32665344

[CR35] Uddin, R., Lee, E.-Y., Khan, S. R., Tremblay, M. S., & Khan, A. (2020). Clustering of lifestyle risk factors for non-communicable diseases in 304,779 adolescents from 89 countries: A global perspective. *Preventive Medicine,**131*, 105955. 31862205 10.1016/j.ypmed.2019.105955

[CR36] van Sluijs, E. M. F., Ekelund, U., Crochemore-Silva, I., Guthold, R., Ha, A., Lubans, D., Oyeyemi, A. L., Ding, D., & Katzmarzyk, P. T. (2021). Physical activity behaviours in adolescence: Current evidence and opportunities for intervention. *The Lancet,**398*(10298), 429–442. 10.1016/S0140-6736(21)01259-9 10.1016/S0140-6736(21)01259-9 PMC7612669 34302767

[CR37] Watkins, D., Hale, J., Hutchinson, B., Kataria, I., Kontis, V., & Nugent, R. (2019). Investing in non-communicable disease risk factor control among adolescents worldwide: A modelling study. *BMJ Global Health,**4*(2), e001335. 10.1136/bmjgh-2018-001335 31139451 10.1136/bmjgh-2018-001335 PMC6509594

[CR38] Webb, T. L., & Sheeran, P. (2006). Does changing behavioral intentions engender behavior change? A meta-analysis of the experimental evidence. *Psychological Bulletin,**132*(2), 249–268. 10.1037/0033-2909.132.2.249 16536643 10.1037/0033-2909.132.2.249

[CR39] Wu, X. Y., Han, L. H., Zhang, J. H., Luo, S., Hu, J. W., & Sun, K. (2017). The influence of physical activity, sedentary behavior on health-related quality of life among the general population of children and adolescents: A systematic review. *PLoS ONE,**12*(11), e0187668. 10.1371/journal.pone.0187668 29121640 10.1371/journal.pone.0187668 PMC5679623

[CR40] Wu, X. Y., Zhuang, L. H., Li, W., Guo, H. W., Zhang, J. H., Zhao, Y. K., Hu, J. W., Gao, Q. Q., Luo, S., Ohinmaa, A., & Veugelers, P. J. (2019). The influence of diet quality and dietary behavior on health-related quality of life in the general population of children and adolescents: A systematic review and meta-analysis. *Quality of Life Research,**28*(8), 1989–2015. 10.1007/s11136-019-02162-4 30875010 10.1007/s11136-019-02162-4

