# Supplementary material for: Adolescent behavioural intentions: Secondary outcomes from a cluster randomized controlled trial of the Health4Life school-based lifestyle modification intervention
Source: Can J Public Health. 2024 Nov 19;116(3):432–45. doi: 10.17269/s41997-024-00955-w (PMC12381305; doi:10.17269/s41997-024-00955-w)

**Supplementary Materials**

1. **Table S.1** Secondary outcomes at baseline by follow-up status of each outcome
2. **Table S.2** Summary of attrition analyses between groups for secondary outcomes
3. **Table S.3**. Summary of model fit estimates for the best fitting unconditional growth models
4. **Table S4.** Subgroup analysis sample sizes
5. **Table S5.** CONSORT 2010 checklist of information to include when reporting a cRCT
6. **Figure S1.** Example slides from the cartoon modules

| **Table S1** Outcomes at baseline by follow-up status of each outcome. | | | | |
| --- | --- | --- | --- | --- |
|  | **0 FUs (N)** | **1+ FUs (N)** | **Odds ratio (95%CI)** | **p** |
| **Alcohol use intentions** | **N = 307** | **N = 6332** | 0.88 (0.69, 1.11) | 0.270 |
| Very unlikely | 60 | 1586 |  |  |
| Unlikely | 28 | 836 |  |  |
| Unsure | 50 | 1326 |  |  |
| Likely | 68 | 1500 |  |  |
| Very likely | 34 | 805 |  |  |
| **Tobacco use intentions** | **N = 305** | **N = 6334** | 0.76 (0.55, 1.06) | 0.105 |
| Very unlikely | 190 | 5059 |  |  |
| Unlikely | 20 | 572 |  |  |
| Unsure | 13 | 270 |  |  |
| Likely | 10 | 71 |  |  |
| Very likely | 3 | 55 |  |  |
| **Physical activity intentions** | **N = 307** | **N = 6332** | 1.00 (0.78, 1.28) | 0.985 |
| Not at all true of me | 8 | 272 |  |  |
| Not very true of me | 18 | 545 |  |  |
| Somewhat true of me | 92 | 2107 |  |  |
| Very true of me | 118 | 3109 |  |  |
| **Screen time intentions** | **N = 312** | **N = 6327** | 1.21 (0.96, 1.53) | 0.112 |
| Not at all true of me | 44 | 917 |  |  |
| Not very true of me | 69 | 1690 |  |  |
| Somewhat true of me | 97 | 2452 |  |  |
| Very true of me | 30 | 950 |  |  |
| **Sleep intentions** | **N = 311** | **N = 6328** | 1.30 (1.03, 1.64) | 0.026 |
| Not at all true of me | 41 | 808 |  |  |
| Not very true of me | 54 | 1310 |  |  |
| Somewhat true of me | 88 | 2116 |  |  |
| Very true of me | 55 | 1775 |  |  |
| **Discretionary beverage intentions** | **N = 309** | **N = 6330** | 1.08 (0.85, 1.36) | 0.529 |
| Not at all true of me | 48 | 1205 |  |  |
| Not very true of me | 43 | 990 |  |  |
| Somewhat true of me | 74 | 1817 |  |  |
| Very true of me | 74 | 2010 |  |  |
| Note. Models are ordinal | | | | |

| Table S2. Summary of attrition analyses between groups for secondary outcomes | | | | | | |
| --- | --- | --- | --- | --- | --- | --- |
|  |  | **Control (N)** | **Intervention (N)** | **Odds Ratio (95%CI)** | | **p** |
| **Alcohol Use intentions** | |  |  | 0.81 (0.64, 1.02) | | .077 |
|  | 0 FUs | 125 | 182 |  |  |  |
|  | 1+ FUs | 2905 | 3427 |  |  |  |
| **Tobacco Use Intentions** | |  |  | 0.81 (0.64, 1.02) | | .074 |
|  | 0 FUs | 124 | 181 |  |  |  |
|  | 1+ FUs | 2906 | 3428 |  |  |  |
| **Physical activity intentions** | |  |  | 0.82 (0.65, 1.04) | | .098 |
|  | 0 FUs | 126 | 181 |  |  |  |
|  | 1+ FUs | 2904 | 3428 |  |  |  |
| **Screen time intentions** | |  |  | 0.81 (0.64, 1.02) | | .073 |
|  | 0 FUs | 127 | 185 |  |  |  |
|  | 1+ FUs | 2903 | 3424 |  |  |  |
| **Sleep intentions** | |  |  | 0.81 (0.64, 1.03) | | .082 |
|  | 0 FUs | 127 | 184 |  |  |  |
|  | 1+ FUs | 2903 | 3425 |  |  |  |
| **Discretionary beverage intentions** | |  |  | 0.80 (0.36, 1.01) | | .061 |
|  | 0 FUs | 125 | 184 |  |  |  |
|  | 1+ FUs | 2905 | 3425 |  |  |  |
| *Note*. FUs = follow-ups. | | | | | | |

**Statistical Analyses:** **Model Fit**

| Table S.3. Summary of model fit estimates for the unconditional mixed effects models | | | |
| --- | --- | --- | --- |
|  | **Time score** | **AIC** | **BIC** |
| **Alcohol_1_** |  |  |  |
|  | Linear | 67242.99 | 67282.86 |
|  | Quad | 67231.84 | 67279.69 |
|  | Categorical | 67229.88 | 67285.70 |
| **Tobacco_2_** |  |  |  |
|  | Linear | 30921.22 | 30961.08 |
|  | Quad | 30881.99 | 30929.82 |
|  | Categorical | 30881.54 | 30937.34 |
| **MVPA_1_** |  |  |  |
|  | Linear | 46054.52 | 46086.41 |
|  | Quad | 46056.48 | 46096.33 |
|  | Categorical | 46051.28 | 46099.10 |
| **Screen time_1_** |  |  |  |
|  | Linear | 56188.42 | 56220.29 |
|  | Quad | 56181.85 | 56221.69 |
|  | Categorical | 56087.29 | 56135.10 |
| **Sleep_1_** |  |  |  |
|  | Linear | 55654.22 | 55694.06 |
|  | Quad | 55655.98 | 55703.79 |
|  | Categorical | 55572.16 | 55627.94 |
| **Discretionary beverages_1_** | |  |  |
|  | Linear | 56499.35 | 565131.22 |
|  | Quad | 56500.57 | 56540.42 |
|  | Categorical | 56422.93 | 56470.75 |
| *Note*. AIC = Akaike information criterion. BIC = Bayesian information criterion. 1 = ordinal logistic mixed effects models. 2 = negative binomial mixed effects regression model. | | | |

Table S4. Sample size of subgroup analysis

|  |  | **Control (N)** | **Intervention (N)** |
| --- | --- | --- | --- |
| Alcohol | Had not consumed a full standard drink* | 2959 | 3499 |
|  | Had consumed a full standard drink | 71 | 110 |
| Tobacco | Had not smoked a cigarette* | 2987 | 3555 |
|  | Had smoked a cigarette | 43 | 54 |
| MVPA | Did not get 7 days MVPA per week* | 2388 | 2823 |
|  | Achieved 7 days MVPA per week | 642 | 786 |
| Screentime | Exceeded 2hrs sedentary recreational screentime* | 2640 | 3135 |
|  | Less than 2hrs sedentary recreational screentime | 390 | 474 |
| Sleep | Didn't meet recommended 9-11 hrs sleep* | 1403 | 1702 |
|  | Slept for 9-11hrs | 1627 | 1907 |
| SSB | Consumed SSB more than once per week* | 1782 | 2297 |
|  | Consumed SSB less than once per week | 1248 | 1312 |

*Subgroup included in sensitivity analyses

**Table S5. CONSORT 2010 checklist of information to include when reporting a cRCT**

| Section/Topic | Item No | Standard Checklist item | Extension for cluster designs | Section |
| --- | --- | --- | --- | --- |
| Title and abstract | | | | |
|  | 1a | Identification as a randomised trial in the title | Identification as a cluster randomised trial in the title | Title |
|  | 1b | Structured summary of trial design, methods, results, and conclusions (for specific guidance see CONSORT for abstracts) | See table 2 | Abstract |
| Introduction | | | | |
| Background and objectives | 2a | Scientific background and explanation of rationale | Rationale for using a cluster design | Introduction |
|  | 2b | Specific objectives or hypotheses | Whether objectives pertain to the cluster level, the individual participant level or both | Introduction |
| Methods | | | | |
| Trial design | 3a | Description of trial design (such as parallel, factorial) including allocation ratio | Definition of cluster and description of how the design features apply to the clusters | Study design and procedure |
|  | 3b | Important changes to methods after trial commencement (such as eligibility criteria), with reasons |  | n/a |
| Participants | 4a | Eligibility criteria for participants | Eligibility criteria for clusters | Study procedure |
|  | 4b | Settings and locations where the data were collected |  | Study procedure |
| Interventions | 5 | The interventions for each group with sufficient details to allow replication, including how and when they were actually administered | Whether interventions pertain to the cluster level, the individual participant level or both | The <Redacted> Intervention and active control |
| Outcomes | 6a | Completely defined pre-specified primary and secondary outcome measures, including how and when they were assessed | Whether outcome measures pertain to the cluster level, the individual participant level or both | Outcomes |
|  | 6b | Any changes to trial outcomes after the trial commenced, with reasons |  | n/a |
| Sample size | 7a | How sample size was determined | Method of calculation, number of clusters(s) (and whether equal or unequal cluster sizes are assumed), cluster size, a coefficient of intracluster correlation (ICC or k), and an indication of its uncertainty | Study design and procedure |
|  | 7b | When applicable, explanation of any interim analyses and stopping guidelines |  | n/a |
| Randomisation: | | | | |
| Sequence generation | 8a | Method used to generate the random allocation sequence |  | Study design and procedure |
|  | 8b | Type of randomisation; details of any restriction (such as blocking and block size) | Details of stratification or matching if used | Study design and procedure |
| Allocation concealment mechanism | 9 | Mechanism used to implement the random allocation sequence (such as sequentially numbered containers), describing any steps taken to conceal the sequence until interventions were assigned | Specification that allocation was based on clusters rather than individuals and whether allocation concealment (if any) was at the cluster level, the individual participant level or both | Study design and procedure |
| Implementation | 10 | Who generated the random allocation sequence, who enrolled participants, and who assigned participants to interventions | Replace by 10a, 10b and 10c | n/a |
|  | 10a |  | Who generated the random allocation sequence, who enrolled clusters, and who assigned clusters to interventions | Study design and procedure |
|  | 10b |  | Mechanism by which individual participants were included in clusters for the purposes of the trial (such as complete enumeration, random sampling) | Study design and procedure |
|  | 10c |  | From whom consent was sought (representatives of the cluster, or individual cluster members, or both), and whether consent was sought before or after randomisation | Study design and procedure |
| Blinding | 11a | If done, who was blinded after assignment to interventions (for example, participants, care providers, those assessing outcomes) and how |  | Study design and procedure |
|  | 11b | If relevant, description of the similarity of interventions |  | n/a |
| Statistical methods | 12a | Statistical methods used to compare groups for primary and secondary outcomes | How clustering was taken into account | Statistical analysis |
|  | 12b | Methods for additional analyses, such as subgroup analyses and adjusted analyses |  | Statistical analysis |
| Results | | | | |
| Participant flow (a diagram is strongly recommended) | 13a | For each group, the numbers of participants who were randomly assigned, received intended treatment, and were analysed for the primary outcome | For each group, the numbers of clusters that were randomly assigned, received intended treatment, and were analysed for the primary outcome | Figure 1 |
|  | 13b | For each group, losses and exclusions after randomisation, together with reasons | For each group, losses and exclusions for both clusters and individual cluster members | Figure 1 |
| Recruitment | 14a | Dates defining the periods of recruitment and follow-up |  | Study design and procedure |
|  | 14b | Why the trial ended or was stopped |  | n/a |
| Baseline data | 15 | A table showing baseline demographic and clinical characteristics for each group | Baseline characteristics for the individual and cluster levels as applicable for each group | Table 1 |
| Numbers analysed | 16 | For each group, number of participants (denominator) included in each analysis and whether the analysis was by original assigned groups | For each group, number of clusters included in each analysis | Figure 1, Table 1, Table 2 |
| Outcomes and estimation | 17a | For each primary and secondary outcome, results for each group, and the estimated effect size and its precision (such as 95% confidence interval) | Results at the individual or cluster level as applicable and a coefficient of intracluster correlation (ICC or k) for each primary outcome | Results Table 2 and Table 3 |
|  | 17b | For binary outcomes, presentation of both absolute and relative effect sizes is recommended |  | n/a |
| Ancillary analyses | 18 | Results of any other analyses performed, including subgroup analyses and adjusted analyses, distinguishing pre-specified from exploratory |  | Results |
| Harms | 19 | All important harms or unintended effects in each group (for specific guidance see CONSORT for harms) |  | n/a |
| Discussion | | | | |
| Limitations | 20 | Trial limitations, addressing sources of potential bias, imprecision, and, if relevant, multiplicity of analyses |  | Discussion |
| Generalisability | 21 | Generalisability (external validity, applicability) of the trial findings | Generalisability to clusters and/or individual participants (as relevant) | Discussion |
| Interpretation | 22 | Interpretation consistent with results, balancing benefits and harms, and considering other relevant evidence |  | Discussion |
| Other information | | | | |
| Registration | 23 | Registration number and name of trial registry |  | Study design |
| Protocol | 24 | Where the full trial protocol can be accessed, if available |  | Study design |
| Funding | 25 | Sources of funding and other support (such as supply of drugs), role of funders |  | Funding statement (supplied separately) |

**Figure S1.** Example slides from the cartoon modules


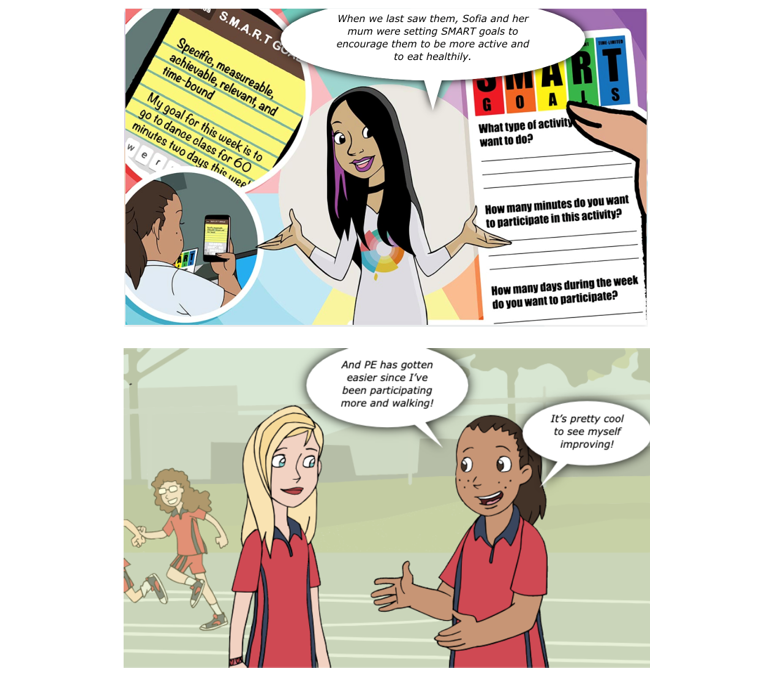

Supplement: Supplementary file 1 — Supplementary file1 (DOCX 418 KB) [file 41997_2024_955_MOESM1_ESM.docx]
